# Lethal and Sublethal Toxicity Comparison of BFRs to Three Marine Planktonic Copepods: Effects on Survival, Metabolism and Ingestion

**DOI:** 10.1371/journal.pone.0147790

**Published:** 2016-01-29

**Authors:** Wenjing Gong, Liyan Zhu, Ya Hao

**Affiliations:** Ocean University of China, College of Marine Life Sciences, Qingdao, Shandong, China; Chinese Research Academy of Environmental Sciences, CHINA

## Abstract

The estuarine planktonic copepods have a wide geographical distribution and commendable tolerance to various kinds of contaminants. The primary aim of the present study was to contrast the impacts of model POPs (TBBPA and HBCD) on three common estuarine planktonic copepods (*Oithona similis*, *Acartia pacifica* and *Pseudodiaptomus inopinus*) and establish a protocol for the assessment of acute toxicity of marine organic pollutants. We first quantified the 96h-LC_50_ (0.566, 0.04 and 0.257 mg/L of TBBPA to the three subjects above respectively and 0.314 mg/L of HBCD to *P*. *inopinus*; all reported concentrations are nominal values). In the sub-lethal toxicity tests, it was turned out that the effects of copepods exposed to TBBPA could product different influences on the energy ingestion and metabolism. Different type of pollutions, meanwhile, could also bring varying degree effect on the target copepods. In general, the indicators (the rate of oxygen consumption, ammonia excretion, food ingestion and filtration) in higher concentration groups showed marked significant difference compared with controls as well a dose-effect relationship. The study also extended the research on the joint toxicity of TBBPA and HBCD based on the survival rate of *P*.*inopinus*. Whether 1:1 concentration or 1:1 toxic level, the research showed synergy effect relative to single exposure conditions. The result indicated that current single ecological testing used for environmental protection activities may underestimate the risk for copepods. It was also demonstrated that short-term sub-lethal experiment could be a standard to evaluate the sensitivity of copepods to POPs.

## Introduction

To reduce the frequency and extent of fire, human usage of flame retardants in industrial goods and everyday life has gradually increased over the last century[1–3]. Flame retardants are classified based on their elements: halogen (includes brominated and chlorinated), phosphorous and antimony-containing[4]. Due to their thermostability and flame retarding effects[5, 6], the brominated flame retardants (BFRs) have become one of the most extensively used polymers around the world[7–9]. As a result of resistance to biodegradation, BFRs are easily accumulated and dispersed in the environment, particularly when not covalently bound[10].

Tetrabromobis-phenol A (TBBPA) is currently one of the most productive BFRs around the world[11]. Global market demand for TBBPA was approximately 170000 tons in 2004, accounting for around 60 percent of the market of BFRs especially in Asia[9, 12]. TBBPA can be used reactively or additively in various types of resin particularly in epoxy resin of printed circuit boards or other acrylonitrile-butadienestyrene polymers[13, 14]. In spite of a relatively short biological half-life and low reactivity, TBBPA can contaminate the environment when released from the product both during and after use[15, 16]. Research has shown that, even if low in water, the solubility of TBBPA can reach 4.16 mg/L at 25 °C[17]. At present, TBBPA commonly exists in many environmental mediums such as soil, water, sediment and air[13]. Zhang P Q et al (2006) measured that the mean concentration of TBBPA in surface water of Chao Lake (Anhui province, China) was 0.52 μg/L, and 21.96~481.80 ng/g (dry weight) in sediment[18]. Tests on seawater showed that the level of TBBPA was up to 39~673 ng/L in Circum-Bohai area of China[19].

1,2,5,6,9,10-Hexabromocyclododecane (HBCD) is a type of non-aromatic brominated cycloalkane and primarily found in polystyrene foam plastic used by the construction construction industry. It is also used for additional applications in other realms such as the coating of textile fabrics, cable, rubber adhesive and the production of unsaturated polyester[20]. HBCD generally includes three diastereomer types used in the industry— α-, β- and γ-HBCD—with proportions of 10–13, 1–12 and 75–89%, respectively[21]. Apart from that of commercial HBCD formulations, many investigations have shown that α-HBCD is the predominant diastereoisomer in biota from both fresh and marine environments[22–24].

Despite low solubility, HBCD can also cause a huge impact in the ocean by its sustained release during manufacturing processes[25]. Earlier survey results showed that the concentrations of α-, β- and γ-HBCD in Winnipeg Lake were 14, 15 and 4 pg/L, respectively, and ranges of 3–31 ng/L and 0.1–25 μg/kg in water and sediment samples respectively in Sweden[26, 27]. What's more, HBCD was detected in 131 of 133 marine mammal models determined by Zegers with a concentration range of 52–9519 n/ng lw[22]. In toxicology experiments, Wang X R et al indicated that the active oxygen in the liver was induced by continuous exposure to 0.5 mg/L HBCD for 5 days[26].

As reported, TBBPA and HBCD show low toxicity in animals[1, 8]. Contrarily, these compounds have high acute toxicity to algae, molluscs, crustaceans and fish[11]; however, little information on the mechanisms involved is available. As HBCD and TBBPA have a high affinity towards apolar media and a strong ability of accumulation, the destructibility on the marine environment must not be overlooked especially the underlying acute and chronic toxicity effects to the aquatic organisms, and gradually become representative of global pollution problems[28, 29].

In marine environments, copepods are considered to play a very important role in the transfer of material and energy between primary producers and higher trophic levels in coastal, shelf, and oceanic waters [30]. Copepods are popular model test organisms to examine the effects of anthropogenic contaminants and environmental factors[31–33]. The six nauplius (larval) and five copepodite (juvenile) stages can be influenced by external environmental factors and thus the ontogenetic development[34–38]. As common species in China’s estuaries, the Calanoid copepods *Acartia pacifica* and *Pseudodiaptomus inopinus* and Cyclopoida copepod *Oithona similis* also generally distribute in coastal water. All the three copepod species can be easily cultured under laboratory conditions, which are likely to be regarded as potential model animals for ecological risk assessment studies.

Currently, international protocols establish norms for the use of nauplii or juveniles of marine copepods in ecotoxicological tests. And a regulated, short-term test is employed for the determination of the lethal effect on adults or copepodites of the copepod species[37]. According to Medina et al. (2002), it is possible to carry out short-term tests based on the complete cycle, since these tests are sensitive and can be more intuitive to evaluate the pollutant levels[38]. Moreover, Gourmelon and Ahtiainen (2007)[39] states that short-term effects on development may be more sensitive than long-term effects on reproductive aspects. As a key link of marine organic matters migrating from primary production to higher trophic levels, the changes on ingestion capacity or metabolic level of copepods could have a substantial effect on the energy flow and circulation of material in marine ecosystems[40]. Therefore, significant importance exists on the basis of acute toxicity experiments to evaluate the effects of TBBPA and HBCD on copepods’ feeding ability and metabolic levels, and accordingly the toxicities of contaminants are analyzed to assess the impacts of these chemicals on the entire marine primary ecosystem[41–44].

The high production volume of TBBPA and HBCD and the presence in aquatic environment suggested that these compounds could affect aquatic organisms. Aiming to contribute to a better understanding of the actue toxicology of TBBPA and HBCD, the primary goals of the present study are: (1) to contrast the acute toxic effects of the contaminants on survival, metabolism and ingestion using three species of common estuarine copepod adults (*O*.*similis*, *A*.*pacifica* and *P*.*inopinus*) as the reference subjects, (2) to analyze the joint-toxicity of TBBPA and HBCD to *P*.*inopinus* in order to appraise the complexity of contaminants in nature. Such data will be useful to demonstrate the versatility of copepods in toxicological research. The study can also provide a dose background of TBBPA and HBCD for any observed effects, and to make data available for risk assessment.

## Materials and Method

### Ethics statement

All procedures using animals were collected from public seawater. We state clearly that no specific permissions were required for these locations/activities and confirm that the field studies did not involve endangered or protected species.

### Copepod maintenance

The copepods (*O*.*similis*, *A*. *pacifica*, and *P*. *inopinus*) tested were collected in 2013 from Qingdao Li-cun River estuary, Cheng-yang District He-tao shrimp ponds, and Laoshan District Nanyao shrimp ponds, respectively. Test organisms were collected using a shallow type III handheld sampling device (77 μm mesh aperture) and transported back to the lab within two hours in 5 L polyethylene plastic buckets. *O*. *similis* and *A pacifica* were held at 20 ± 1°C and 30 ‰ salinity, while *P*. *inopinus* was held at 15 ± 1°C and 15 ‰ salinity. Fresh natural seawater used for holding was collected from Qingdao Lu Xun Park and filtered to 0.45 μm. The pH and dissolved oxygen of the seawater were 8.0 ± 0.3 and 5.68 ± 0.22 mg/L. All three copepod species were kept under a 12:12 h light:dark cycle in 1000 or 2000 mL glass beakers with a density of less than 2 ind/mL. All the toxicity testing conditions were the same as described above for copepod cultures.

Algae *Isochrysis galbana*, *Chlorella vulgaris*, and *Chaetoceros muelleri* were obtained from the College of Fisheries, Ocean University of China. A 5:3:2 mixture ratio of the three algae was provided as daily food at a concentration of (5.5 ± 0.5) ×10^5^ cells/mL. While the toxicological test used *I*. *galbana* as the single food at a concentration of 4 × 10^5^ cells/mL. The microalgae were all cultured at 20 ± 1°C and 25 ‰ salinity in the f/2 medium with sterile twice filtered (0.45 and 0.22 μm) seawater media.

### Test chemicals

Both TBBPA and HBCD were purchased from J&K Chemical Reagent Co., Ltd. (solid powder, China, >95% pure). According to research reported by Hutchinson TH et al.(2006)[45], dimethyl sulfoxide (DMSO) was used as a solvent in each concentration treatment with a final volume-to-volume ratio of 0.05% for adult acute toxicity.

### 96h acute toxicity tests

Previous research in our lab has been conducted to determine the HBCD value on *P*. *inopinus* (Table 1). Standard 96h acute toxicity TBBPA tests were carried out with the three species of copepod adults. The concentration gradients were set up according to a certain common ratio with the mortality less than 10% in the lowest dose and greater than 90% in the highest. There were three replicates per concentration with 15 randomly-selected copepods in each test chamber (100 mL beaker). Each test included positive (filtered seawater) and solvent (DMSO) controls. In DMSO groups, solvents were employed in equal concentrations to match their levels in the highest dose. The copepods were then incubated in an illumination incubator for 96h (feeding *I*. *galbana*) and the test solutions (>50% of the working volume) were renewed once after 48h exposure. Mortality was checked every 24h whereby a copepod was recorded as dead when its urosome was at a right angle to the prosome[46].

**Table 1:** LC_50_ and 95% confidence intervals (CI) for adult *P*.*inopinus* exposed to HBCD (1,2,5,6,9,10-Hexabromocyclododecane) in 96h acute toxicity test at 15°C.

Chemical	LC_50_ (μg/L)	95% confidence interval (μg/L)
HBCD	313.877	277.342–361.851

HBCD was dissolved in dimethyl sulfoxide.

### Sub-lethal toxicity tests of energy budget

Based on the acute toxicity testing, a series of TBBPA concentration gradients (1.25, 2.5, 5, 10 and 20% of 96h-LC_50_) were set on energy budget tests. Because there was no difference between the seawater and the solvent control treatments in either acute tests, this section of experiments only used solvent groups as controls in which concentration was equal to its level in the highest dose treated groups. Meanwhile, the test selected *P*.*inopinus* as the subject to contrast the discrepancies between different contaminants on energy intake and expenditure.

#### Metabolism test

Referring to the specification for marine monitoring——Part 4: seawater analysis (GB17378.4–2007)[47], the iodometry and indophenol blue photometric methods were used to determine the variance of oxygen consumption and ammonia excretion rate, respectively. Both sets of experiments were performed on the same batch of iodine bottles (110 mL approximately). A 2000 mL beaker was cultured in advance and the copepods were collected at the same time to assure developments were closely synchronized. Every 30 copepod adults were randomly transferred to iodine bottles with the corresponding test solution, with triplicates for each concentration. The experiment was continued up to 48h, and then opened quickly to remove the upper layer solution (75 mL approximately) to a reagent bottle. Then the manganese chloride solution and alkaline solution of potassium iodide were injected immediately with 1 mL each, and subsequently stoppered bottles were used to shake upside-down. After 1h’s standing, the oxygen level was then monitored with alkali burette. Furthermore, the remaining solution of each iodine bottle was used to evaluate the quantity of ammonia, which was determined by the ultraviolet spectrophotometer. The oxygen consumption and ammonia excretion rate percentages of adults were calculated as:

$$R_{o}=\frac{0.7\times 1000\times (C_{0}-C_{i})\times V}{n\times t}$$ (1)

Where *R*_o_ is the oxygen consumption rate (μL/ind·h); C_0_ and C_i_ are the dissolved oxygen (DO)concentration (mg/L) of control and treatment groups, respectively; V is represented as the volume of the solution in the test (L); n is the count of the subjects (ind); t is the experiment period (h); 0.7 is the oxygen conversion coefficient which translated from mass to volume.

$$R_{A}=\frac{1000\times (N_{0}\;\text{-}\;N_{\text{i}})\times V}{n\times t}$$ (2)

Where *R*_A_ is the ammonia excretion rate (μg/ind·h); N_0_ and N_i_ are the concentration of ammonia-nitrogen (mg/L) of control and treatment groups, respectively; V is represented as the volume of the solution in the test (L); n is the count of the subjects (ind); t is the experiment period (h).

#### Ingestion test

Five test concentrations and one solvent control were carried out. Triplicates were set up for each treatment with 30 adult copepods at 15 (for *P*. *inopinus)* or 20 °C (for O.*similis* and *A*. *pacifica*). To start the feeding test, copepods after a 24h starvation treatment were put in a 75mL plastic bottle filled with test solution. *I*.*galban* in the logarithmic phase (diluted to 4×10^5^ ind/mL) was served as the unitary composition to evaluate the feeding rate. The bottles were completely sealed and placed into a feeding wheel gearing with the speed of 1 r/min to implement a 48h feeding test in a dark environment. Ruger reagent was used to fix the algal cells remaining in the bottles at the end of the test, and the counting process was performed with a standard blood count board under an optical microscopes (OLYMPUS, BH-2). The ingestion of copepods was determined according to the method described by Frost (1972)[48]. The formulae for converting algal cells into the filtration and ingestion rate percentages were calculated as:

$$F=\frac{V}{N}\cdot \frac{\ln c_{t}-\ln c_{tf}}{t}$$ (3)

$$G=F\cdot \frac{{\text{c}}_{tf}-c_{0}}{\ln c_{tf}-\ln c_{0}}$$ (4)

Where F is the filtration rate (mL/ind·h) and G is the ingestion rate (cells/ind·h);V is the volume of the solution in the test (mL);N is the count of the subjects (ind); c_0_, c_i_ and c_tf_ are, in order, the initial concentration of algal in each groups, the final concentration in control groups and the final concentration in each treatment group, respectively (cells/mL); t is the experiment period (h).

### Joint toxicity tests of TBBPA and HBCD

Based on the independent 96h acute toxicity test of *P*.*inopinus* exposed to TBBPA and HBCD, the additive index method was used to evaluate the joint toxicity reported by Marking L L(1977)[49]. There were two mixed modes (1:1 concentrations and 1:1 toxic levels of TBBPA and HBCD) adopted in the experiment to measure the joint toxic damage degrees. Two tests were setup containing 10 and 8 experimental groups (2 controls and the rest were treatments) with 3 replicates, respectively. Each replicate was made available for 15 copepod adults. 96h joint toxicity by TBBPA and HBCD in two mixture ways were calculated as:

$$S=\frac{A_{\text{m}}}{A_{1}}+\frac{B_{m}}{B_{1}}$$ (5)

Where S is the sum of the toxic additive effect of contaminants exposed on the testing organisms; A_1_ and B_1_ are the independent LC_50_ in the single toxic exposure, respectively. The calculations is converted from S to additive index (AI) by the following formula:

$$AI=\left\{ \begin{array}{l} \frac{1}{S}-1.0,\;S\leq 1\\ (-1)S+1.0,\;S>1 \end{array}\right.$$ (6)

The additive index (AI) is used as the evaluation standard of the combined effects of pollutants: the effect between the two contaminants is synergistic, antagonistic or additive action when AI is >0, = 0 or <0, respectively.

### Statistical analyses

Data is presented as the mean ± standard error (SE). And the differences between the groups were analyzed by one-way analysis of variance (one-way ANOVA). If significant (P < 0.05) differences were found by the ANOVA test, the LSD test was used to determine pair-wise differences between means. All statistical analyses were carried out using SPSS version 16.0.

## Results

### 96h acute toxicity of TBBPA and HBCD

**Fig 1:**
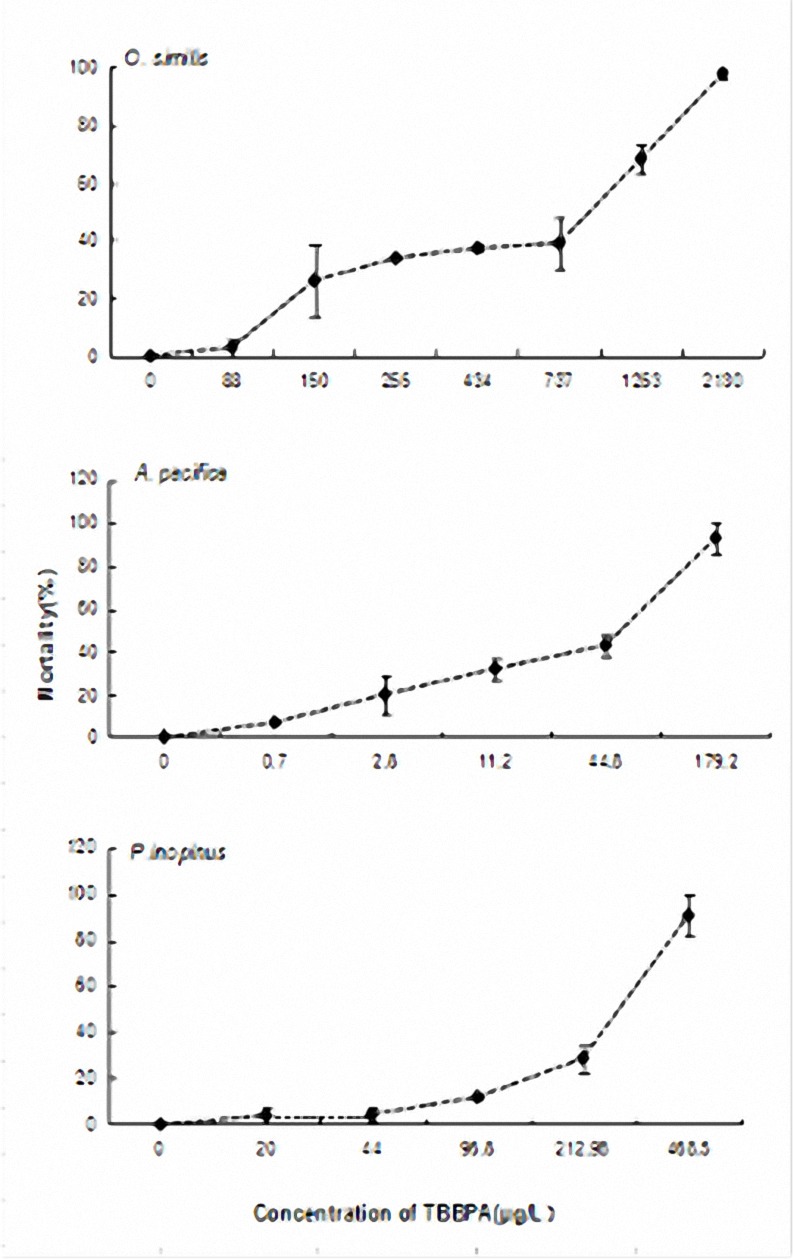
Mortality of the target copepod adults exposed to TBBPA (Tetrabromobis-phenol A) during 96-h acute toxicity tests. The concentrations are set as: control—copepods exposed to untreated seawater, the lowest dose—the mean mortality lower than 10%, the highest dose—the mean mortality higher than 90%. Data is mean ± SE (n = 3).

The mean survival rates of the three species of copepods in the seawater and DMSO controls were 100 and 99.1 ± 0.3%, implying that there was no solvent effect. With increasing TBBPA concentration, the survival decreased significantly. The results indicated that the mortality had a dose response with increasing TBBPA concentration. The calculated 96h-LC_50_ of *O*.*similis*,*A*.*pacifica* and *P*.*inopinus* was 0.566, 0.04 and 0.257 mg/L, respectively(**Fig 1**). Previous internal studies generated an acute 96h-LC_50_ of 0.314 mg/L to *P*.*inopinus*(**Table 1**).

### Effects on energy budget (cross-species comparison)

#### Metabolism

**Fig 2:**
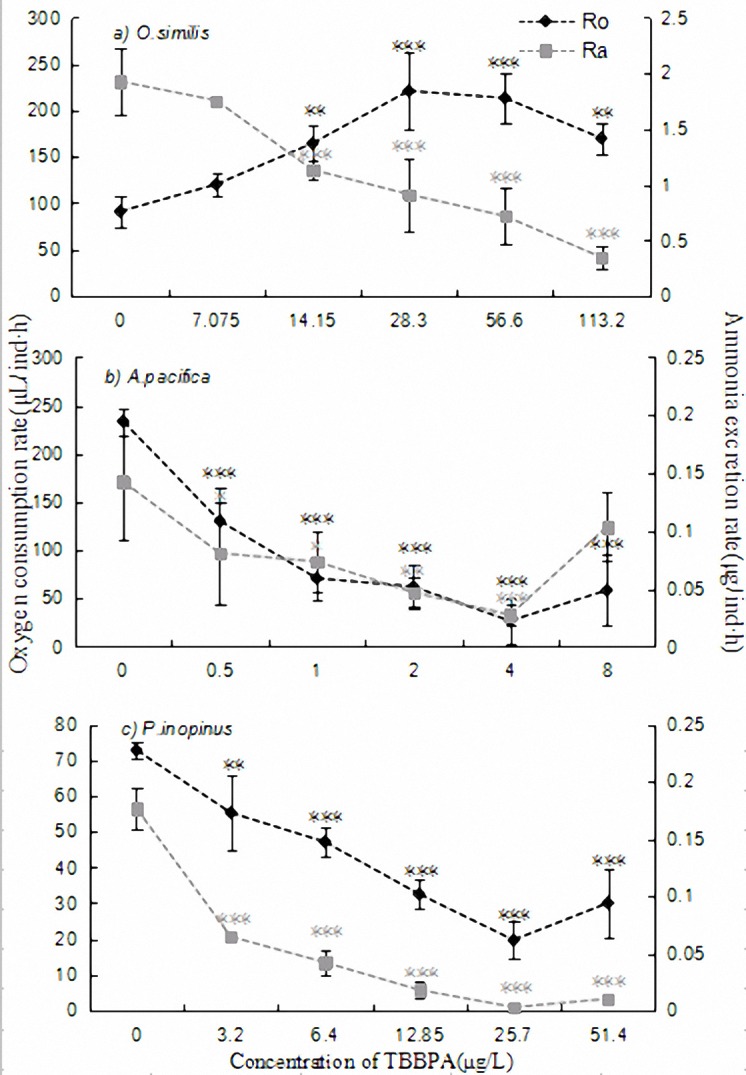
Oxygen consumption and ammonia excretion rates of three target copepod adults exposed to different concentrations of TBBPA during the experimental period. The concentrations are set as: control—copepods exposed to untreated seawater, the treatments—copepods exposed to 1.25, 2.5, 5, 10 and 20% of 96h-LC_50_, respectively. Significant difference from the control treatment is indicated by *p<0.05,**p<0.01 and ***p<0.001, respectively. Data is mean ± SE (n = 3).

As shown in **Fig 2A**, respiration rate (R_o_) of *O*.*similis* in each treatment group exposed to TBBPA was higher than that of the controls, and the change curve was an inverse U-shape in which the mean maximum 220.99 μL/ind·h (p<0.001) appeared at 28.3 μg/L treatment. Contrary to R_o_, the ammonia excretion rate (R_a_) of *O*.*similis* linearly decreased with the increasing of the concentrations and the minimum value (0.3487 μg/ind·h, p<0.001) appeared at the highest dose (113.2 μg/L, 20%96h-LC_50_). Except the lowest dose, each treatment group showed a significant difference comparing with the controls (p<0.01).

*A*.*pacifica* and *P*.*inopinus* decreased their respiration rate and metabolic activity in the presence of TBBPA (**Fig 2B and 2C**). Unlike *O*.*similis*, the R_o_ and R_a_ of the two subjects were restrained with a dose effect relationship in which minimums were both in 10% 96h-LC_50_ groups, and the change of R_a_ was largely moving in tandem with R_o_. Besides the highest dose to *A*.*pacifica*, the various data of each experimental group obviously lower than those in the controls, and the difference was statistically significant (p<0.05).

#### Feeding

**Fig 3:**
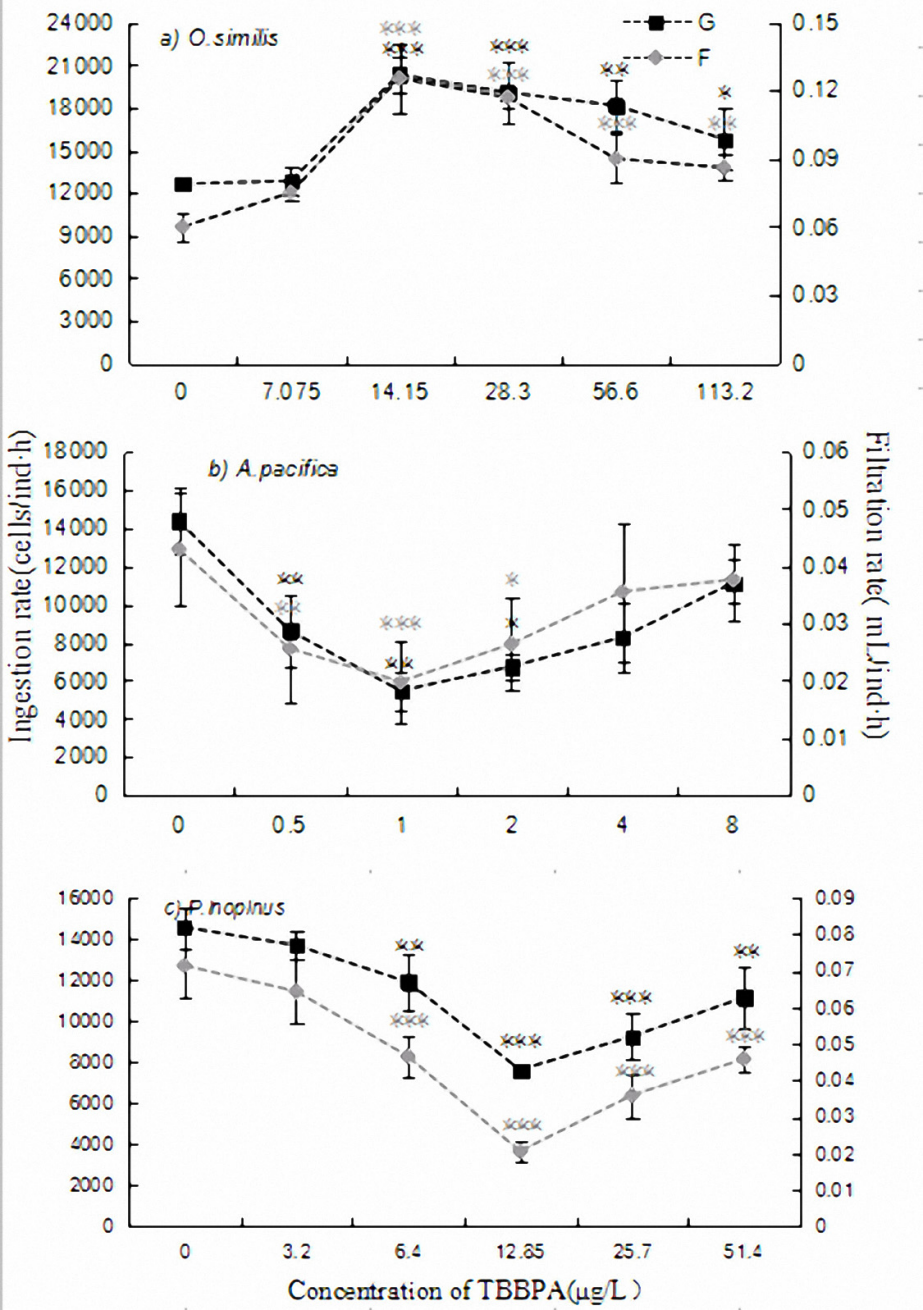
Ingestion and filtration rates of the target copepod adults exposed to different concentrations of TBBPA during the experimental period. The concentrations are set as: control—copepods exposed to untreated seawater, the treatments—copepods exposed to 1.25, 2.5, 5, 10 and 20% of 96h-LC_50_, respectively. Significant difference from the control treatment is indicated by *p<0.05,**p<0.01 and ***p<0.001, respectively. Data is mean ± SE (n = 3).

The results showed that with TBBPA concentration enrichment, the filtration and ingestion rates of *O*.*similis* both rose at beginning then declined in the experimental composition range (**Fig 3A**). The maximal value occurred in the same dosage group (2.5% 96h-LC_50_) and the estimates of which were 20318.15 cells/ind·h and 0.1253 mL/ind·h, respectively. The indexes were differentiated between treatments and normal (p<0.05) besides the minimum concentration.

Instead, filtration and ingestion rates of *A*.*pacifica* and *P*.*inopinus* adults on *I*.*galban* were lower in TBBPA treatment over 48h sub-lethal toxicity test (**Fig 3B and 3C**). The tendencies of the two indexes were equally aligned in which both a U-shaped curve occurred. The mean values 5462.01 cells/ind·h and 0.0198 mL/ind·h of *A*.*pacifica* were the lowest in 2.5% 96h-LC_50_ treated group, and the minimum 4620.78 cells/ind·h and 0.0115 mL/ind·h of *P*.*inopinus* at 5% 96h-LC_50_, respectively. Significant differences were detected predominantly in lower levels (1.25, 2.5 and 5% 96h-LC_50_) of *A*.*pacifica* and intermediate levels (2.5, 5, 10 and 20% 96h-LC_50_) of *P*.*inopinus* for both filtration and ingestion rates.

### Effects on energy budget (compound toxicity comparison)

Synthesized the sensibility to TBBPA and the growth status under laboratory conditions of the three copepod adults, the test selected *P*.*inopinus* as the subject to contrast the effects of TBBPA and HBCD on energy budget. The results showed that the variation tendency of metabolic and feeding processes were essentially the same as those triggered by these two compounds.

#### Metabolism

**Fig 4:**
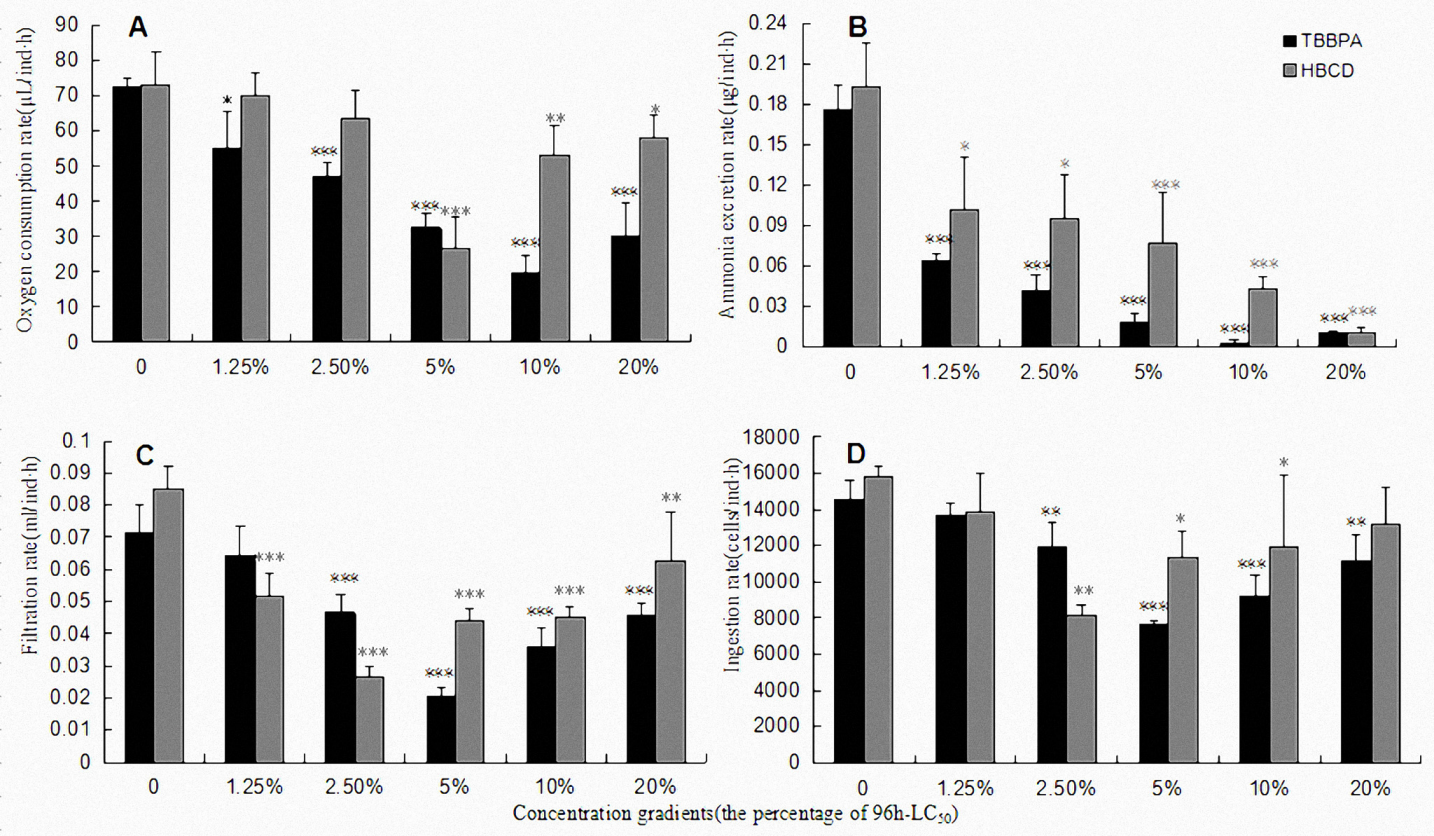
Toxicity comparison between TBBPA and HBCD on the energy budget of *P*. *inopinus*. A, B, C, D represents the rate of oxygen consumption, ammonia excretion, filtration and ingestion, respectively. Gray bars represent the effect of HBCD and black bars for TBBPA. Significant difference from the control treatment is indicated by *p<0.05,**p<0.01 and ***p<0.001, respectively. Data is mean ± SE (n = 3).

The overall influence on the metabolism of *P*.*inopinus* was calculated as the respiration and excretion rate in which were both restrained in varying degrees under alone stress of TBBPA or HBCD (**Fig 4A and 4B**). The energy expended on respiration and excretion of copepod adults in all TBBPA groups decreased more than did controls and showed a significant dose-effect correlation (p<0.01). Compared with TBBPA, *P*.*inopinus* showed stronger adaptability and tolerance to HBCD in low concentrations. The high level exposure to HBCD, likewise, could significantly reduce metabolic rates (both R_o_ and R_a_) of *P*.*inopinus* (p<0.05). Nevertheless, R_o_ was not a significant discrepancy in the low dose of HBCD compared with blank (p>0.05). It was also noted that excretion rate maybe more sensitive than respiration rate for detecting short-term exposure to HBCD.

#### Feeding

**Fig 4C and 4D**revealed the impacts on feed-taking capacity of *P*.*inopinus* treated with sub-lethal concentration of the two kinds of POPs (taking *I*.*galban* as food). It showed that TBBPA and HBCD could both inhibit the rate of ingestion or filtration in certain concentrations with a dose-dependent manner. The changeable tendencies of the two measures were identical in a U-shaped curve. The minimums of ingestion and filtration rates with the action of TBBPA and HBCD were shown separately in the level of 5% and 2.5% 96h-LC_50_ treated. Both of the two POPs could cause prominent toxic effects on food-intake which almost each intermediate dose treatment or above showed a significant difference on *F* and *G* (p<0.05) than those in controls.

### Joint toxicity of 96h mortality

**Fig 5:**
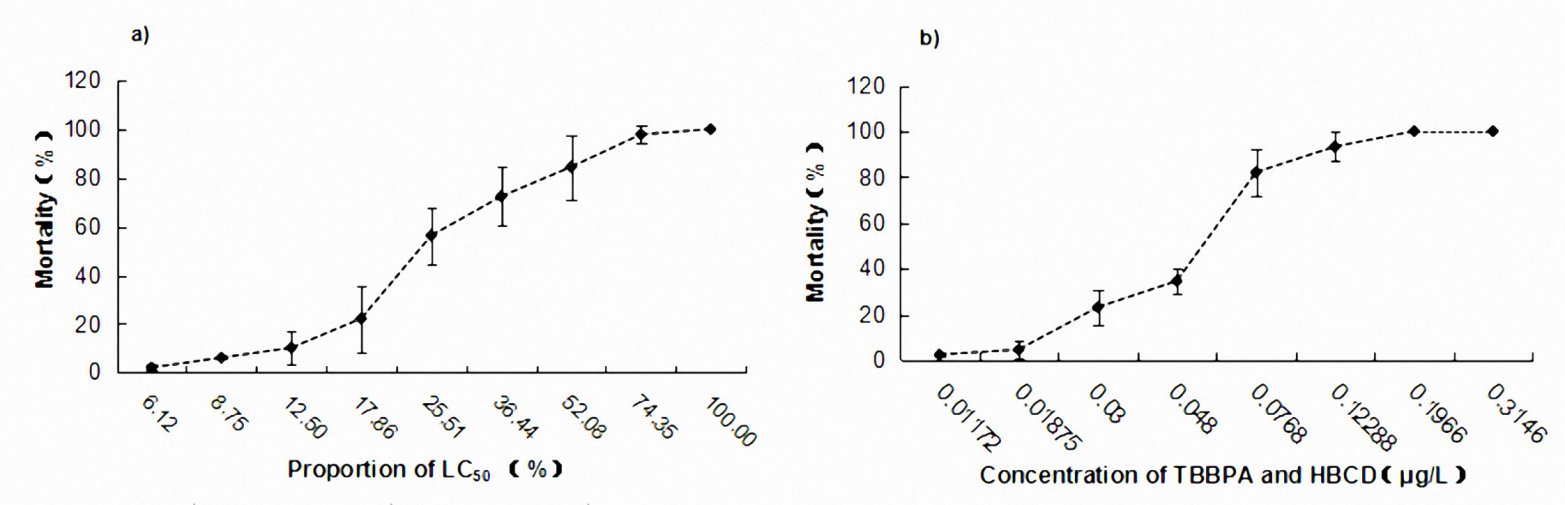
Mortality of *P*.*inopinus* exposed a)virulence 1:1 and b) concentration 1:1 of TBBPA and HBCD during 96h joint toxicity tests. Testing content and methods according to the two mixed modes that 1:1 concentrations or 1:1 toxic levels reported by Marking L L(1977). The concentrations are set as: the lowest dose—the mean mortality lower than 10%, the highest dose—the mean mortality higher than 90%. Data is mean ± SE (n = 3).

There were two types of concentration ratio mixing methods (1:1 concentrations and 1:1 toxic levels) demonstrated at the identical experimental conditions to find out the joint toxic effects of TBBPA and HBCD. The mortality of *P*.*inopinus* was increased with the rising of the concentration and the prolonging of treating time (**Fig 5**).The results indicated that whether 1:1 concentration or 1:1 toxic levels, the contaminants showed a synergy effect compared to single exposure condition.

## Discussion

The acute effects of TBBPA on three species of copepods have shown different expression by contrasting the 96h-LC_50_. Wherein *O*.*similis* exhibited the highest resistance to TBBPA and the most sensitive species was *A*.*pacifica*. Analogously, comparing with Calanoida, previous studies have also shown that the copepods belong to Cyclopoida could demonstrate higher PAHs-tolerance. Barata et al.(2002; 2005)[50,51]found that the 48h-LC_50_ of *Oithona davisae* exposed to naphthaline was 7190.34 μg/L, which value was detected by using *Paracartia grani*[52] or *Pseudodiaptomus poplesia*[53] as the subject was 2535 or 4589.34 μg/L, respectively. In this study, however, even though both the members of Calanoida, there exists obvious differences between *P*.*inopinus* and *A*.*pacifica* in the acute toxicity effects. Based on the research harvest of life history strategies of marine calanoid copepods[54,55], our study speculates that the spawning method may affect the tolerance to contaminants. As an ovigerous species, *P*.*inopinus* carries its eggs in sacs attached to the genital opening until hatching. Thus, females may accumulate harmful substances in the oocyst and separate it off. As *A*.*pacifica* spawn their eggs freely into the water column (broadcast spawners), the toxic level is relatively high. Other research in our lab has calculated that the 96h-LC_50_ of *P*.*inopinus* exposed to HBCD was 0.314 mg/L, which was very close to the value of TBBPA. The researchers speculate that similar physics and chemistry properties may result in an accordant acute toxic effect.

In the absence of a fixed respiratory organ, the gas exchange of copepods is mainly through the body surface and assistant function by using the first and second maxillas to enhance the oxygen transfer during feeding (**S1 Fig**)[56]. Accordingly, it can be concluded that the filter-feeding copepods may take in more harmful substances than those of sarcophagous or parasitical and hence the impacts on the process of respiration are more apparent. In the present study, *O*.*similis* was an omnivore yet *A*.*pacifica* and *P*.*inopinus* were both filter feeders[57,58]. Thus, the respiratory rates of the latter two species constantly decreased with the increasing exposure to pollutants. In contrast, respiration remained obviously higher than the control group by applying *O*.*similis* as the subject.

Consistent with TBBPA, the R_o_ of *P*.*inopinus* contaminated by HBCD was similar to show a trend from a decrease to an increase. Our study concluded that the effects of *P*.*inopinus* exposed to both the two kinds of man-made contaminants on respiratory rate were largely similar. In our previous studies, we have demonstrated the range and sensitivity of other copepod species to POPs. The studies indicated that R_o_ would establish a set of changes with the alteration of contaminants and copepod species. Wherein as a species of filter feeding[59,60], the respiratory rate of *Calanus sinicus* was constantly influenced by negative effects as a result of TBBPA exposure rising[61]. And as an omnivore[62], there was no significant impact on the respiratory rate of *Tigriopus japonicus* exposed to BDE-47 which somehow had been even improved and strengthened obviously in an appropriate dose[63].

The excretory organ of copepods has a simpler structure which contains a couple of irregular curved pipes to connect the stomach and the base of the second maxilla (**S1 Fig**). Various kinds of environmental factors, therefore, can exert direct influence on the metabolic rate of copepods[64]. In the present research, the R_a_ of three target copepods exposed to the contaminants was inhibited in different degrees. Metabolic rate of *O*.*similis* in each treatment was a magnitude greater than that of the other two copepod species even if the change trends influenced by the POPs were roughly consistent. This was unlike the previous studies, however, which sought to reduce the toxicity by increasing the metabolic rate considerably. For instance, R_a_ of *Eurytemora pacifica* and *T*. *japonicus* exposed to BDE-47 both rose at a certain degree[63]. The test indicated that there were different mechanisms could be used to cope with the changing of environmental conditions[64]. Thus, the results can be concluded that the three target copepods may generate a strong inhibited reaction in the metabolic processes caused by sensitive to this type of pollutants.

Prey and ingestion are both the key factors in determining the zooplankton population dynamics[58]. Recent studies show a linear relationship between the ingestion rate of copepods and the microalgae concentration[65, 66]. The ingestion rate of copepods could rise with the microalgae concentration increasing until a threshold was reached[67]. According to daily culture conditions, 4×10^5^ ind/mL was designed as the optimum mean feeding concentration of these three adult copepods in the present study. It was clear that ingestion abilities of target copepods were subjected to different levels of inhibiting action except *O*.*similis* and the changing trends presented down-to-up. Moreover, the test was shown that the variation tendency of ingestion ability was approximately the same as the respiratory rate. The conclusions were putatively drawn that the feeding habit was one of the most crucial factors to evaluate the impacts of POPs on respiration and energy intake, which could directly affect the experiment result. In this study, as both *A*.*pacifica* and *P*.*inopinus* were filter feeders, thus slowing down the rate of respiration and ingestion was an effective countermeasure to reduce actual consumption of the contaminants. *O*.*similis*, conversely, as a omnivore, which respiratory and ingestion rate offered a trend of up-to-down when exposed to the pollutants. It was speculated that there might be a deferred response pattern before the contaminant concentration reaching a certain level that was sufficient to threaten the whole respiratory or ingestion system.

As there was a similarity between the two contaminants in the single toxicity, two types of concentration ratio mixing methods (1:1 concentrations and 1:1 toxic levels) were demonstrated to evaluate the potentially toxic combinations of TBBPA and HBCD. The results showed that the combined toxicity was synergy in whatever methods. According to the international development tendency of POPs[68], these pollutants can act together to appear in a more alarming additive or synergistic effect (even up to 1,000 times more than acting alone) than each individual contaminant that exists in the environment. Recently, Lu D Y et al.(2003)[69] have studied the toxicity of synergy effects and its mechanism in the organisms. They speculated that the joint toxic effects could work in three different ways. Firstly, there might exist multiple binding sites on the receptor monomers, and different contaminants could combine with their respective sites to cause various damage to the organisms. Secondly, the hormone receptors could play a role by the form of homodimer, and therefore some POPs might competitively inhibit the binding ability of estrogen combined with a single monomer. Thirdly, the POPs could associate with other different proteins, and combine with the endoplasmic reticulum to the form of heterodimer which could improve the efficiency of transcription and then might exhibit synergistic effects.

According to the results of joint toxicity, it is presumed that TBBPA can increase total free radicals generation in the organisms which can induce the production of reactive oxygen species (ROS) and may result in significant damage to cellular constituents [70]. Then the toxicity effect of HBCD could be amplified.

## Conclusion

In summary, both TBBPA and HBCD can lead to different influence on survival, metabolic processes and ingestion activities of the target copepods. The results showed that short-term test can be qualified as a new method to estimate the potential risk of POPs in a practical, easy, low-cost project with both lethal and sub-lethal endpoints exhibiting an acceptable degree of variation[37]. Meanwhile, contaminants showed synergy effect compared to single exposure condition in the joint toxicity tests. It is speculated that even relatively low levels of POPs in the natural marine environment could cause potentially great implications by the combined effect of numerous compounds. Under laboratory conditions, high fecundity and POPs-sensitive make *P*.*inopinus* deserving of consideration as a supplement to existing model species after further validation for its use in evaluating local contamination status. Furthermore, this model species would meet the needs for marine pollution monitoring and environmental risk assessment, particularly in China's continental sea and western Pacific coastal regions.

## Supporting Information

S1 Fig The morphological structure of Calanoida males (ventral view). (DOC)
