# Supplementary figures and images for: Lethal and Sublethal Toxicity Comparison of BFRs to Three Marine Planktonic Copepods: Effects on Survival, Metabolism and Ingestion
Source: PLoS One. 2016 Jan 29;11(1):e0147790. doi: 10.1371/journal.pone.0147790 (PMC4732688; doi:10.1371/journal.pone.0147790)

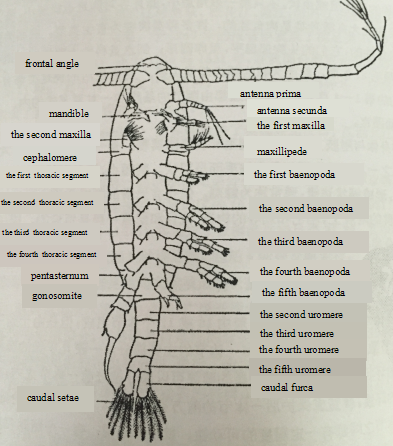


**S1 Fig. The morphological structure of Calanoida males (ventral view)**

Supplement: S1 Fig — (DOC) [file pone.0147790.s001.doc]
